# Folate and B12 Levels Correlate with Histological Severity in NASH Patients

**DOI:** 10.3390/nu10040440

**Published:** 2018-04-02

**Authors:** Mahmud Mahamid, Naim Mahroum, Nicola Luigi Bragazzi, Kasem Shalaata, Yarden Yavne, Mohammad Adawi, Howard Amital, Abdulla Watad

**Affiliations:** 1 Endoscopy Unit, Nazareth Hospital EMMS, 16100 Nazareth, Israel; mahmudmahamid@yahoo.com; 2 Azrieli Faculty of Medicine, Bar-Ilan University, 13195 Safed, Israel; sh.kasem@hotmail.com; 3 Department of Medicine ‘B’, Sheba Medical Center, Tel-Hashomer, Israel, Sackler Faculty of Medicine, Tel-Aviv University, 52621 Tel-Aviv, Israel; naim.mahroum@gmail.com (N.M.); yavneyarden@gmail.com (Y.Y.); Howard.Amital@sheba.health.gov.il (H.A.); 4 School of Public Health, Department of Health Sciences, University of Genoa, 16132 Genoa, Italy; robertobragazzi@gmail.com; 5 Internal Medicine Department, Nazareth Hospital EMMS, 16100 Nazareth, Israel; 6 Ziv and Padeh Medical centers, 13195 Safed, Israel; Limak@012.net.il

**Keywords:** nonalcoholic fatty liver disease, NAFLD, nonalcoholic steatohepatitis, NASH, vitamin B9, folate, vitamin B12

## Abstract

Background: The correlation between abnormal vitamin serum levels and chronic liver disease has been previously described in literature. However, the association between the severity of folate serum levels (B9), vitamin B12 and nonalcoholic steatohepatitis (NASH) has not been widely evaluated. Therefore, the aim of this study was to investigate the existence of such a correlation in a cohort of NASH patients. Methods: All patients aged 18 years and older who were diagnosed with biopsy-proven NASH at the EMMS hospital in Nazareth during the years 2015–2017 were enrolled in this study. Data regarding demographic, clinical and laboratory parameters was collected. Patients with other liver diseases were excluded. Results: Eighty-three NASH patients were enrolled during the study period. The mean age was 41 ± 11 years and the majority of patients were male. Mean values of folate and B12 were 9.85 ± 10.90 ng/mL and 387.53 ± 205.50 pg/mL, respectively. Half of the patients were presented with a grade 1 steatosis (43.4%), a grade 2 fibrosis (50.6%) and a grade 3 activity score (55.4%). The fibrosis grade was significantly correlated with low folate levels on multivariate analysis (*p*-value < 0.01). Similarly, low B12 levels were significantly associated with a higher fibrosis grade and NASH activity (*p*-value < 0.001 and *p*-value < 0.05 respectively). Conclusion: Our study demonstrated a statistically significant correlation between low levels of folate and vitamin B12 with the histological severity of NASH. These findings could have diagnostic and therapeutic implications for patient management and follow-up.

## 1. Introduction

Nonalcoholic fatty liver disease (NAFLD) is defined as the presence of liver steatosis, as demonstrated on imaging or histology, in patients who ingest less than 20 mg of alcohol per day [1,2]. It is the most prevalent liver disease worldwide, with an estimated global prevalence of 25.24% (95% confidence interval (CI) 22.10–28.65), of which the highest rates are found in the Middle East and South America [3,4]. Different metabolic co-morbidities are often associated with NAFLD, including central obesity, insulin resistance, type 2 diabetes mellitus, hyperlipidemia, hypertension, and metabolic syndrome [4,5].

NAFLD is a wide spectrum of disorders ranging from a simple form of steatosis to a progressive form known as nonalcoholic steatohepatitis (NASH), which is characterized by hepatocellular injury and inflammation [6]. Furthermore, NAFLD is considered to be an important cause of cryptogenic cirrhosis [7]. According to a recently published meta-analysis, the mean annual rate of fibrosis progression in NASH is 0.09 (95% CI 0.06–0.12), whilst the overall fibrosis progression proportion is 40.76% (95% CI 34.69–47.13) [4]. Treatment of NASH continues to be challenging for physicians as there is no approved effective pharmacotherapy for this disorder and life style modification remains the mainstay of therapy for these patients [2,8,9].

Vitamin deficiency/hypovitaminosis is a general term for a wide-spread condition which has reached epidemic proportions in developing and developed countries alike, mainly due to lifestyle and dietary habits. Although sub-clinical hypovitaminosis can occur in well-nourished subjects, it is most often the result of limited dietary intake or vitamin malabsorption [9,10]. Several studies have shown that chronic liver diseases are associated with lower levels of vitamins such as vitamin D, E, B6, and A [11,12,13,14]. Moreover, a trial therapeutic combination of vitamins E and A as a treatment for NASH was found to minimize oxidative stress damage and to curb cirrhosis formation processes [15].

Despite the indications in the literature regarding the association between chronic liver diseases and hypovitaminosis, little is known about a specific correlation between hypovitaminosis and NASH severity. Thus, in this study we sought to investigate potential correlations between folate and B12 serum levels with NASH severity, as defined in terms of the fibrosis grade and activity, in a geographical area characterized by a significant epidemiological burden of NASH. We chose to focus on these vitamins because the relationship between their serum levels and fibrosis severity has been scarcely reported. More specifically, we expected to find a negative correlation, in analogy with what was found for other vitamins.

## 2. Material and Methods

### 2.1. Ethical Approval 

The current study received ethical approval from the local hospital ethical committee and was conducted according to the Helsinki declaration and its subsequent amendments. The data was coded in order to preserve the anonymity of the patients. Informed consent was waived because of the non-interventional design of the study.

### 2.2. Patients Selection

All patients diagnosed with biopsy-proven NASH at the EMMS Nazareth Hospital (located in Nazareth, Israel) between the years 2015 to 2017 were considered potentially eligible and enrolled in the study. Furthermore, patients were included in the present study if (i) age > 18 years old; and (ii) they had no history of alcohol abuse (their weekly ethanol consumption was less than 210 gm).

Patients with other hepatic pathology or autoimmune phenotypes (such as alcoholic liver disease, drug-induced liver injury, autoimmune hepatitis, viral hepatitis, cholestatic liver disease and metabolic/genetic liver disease) were excluded using specific clinical, laboratory, radiological and/or histological criteria/tests (serology of viral hepatitis A, B, and C, autoimmune markers including ANA, anti-LKM, anti-smooth mussels protein electrophoresis, immune electrophoresis, metabolic markers such as serum ceruloplasmin, 24-h urine collection for copper, ferritin, iron, transferrin saturation, TSH, HbA1c and alpha-1 antitrypsin).

Extracted data included socio-demographic variables (such as age and gender), body mass index (BMI), fat volume, histopathological biopsy results, serum levels of alanine transaminase (ALT), aspartate transaminase (AST), low-density lipoprotein cholesterol (LDL), high-density lipoprotein cholesterol (HDL), triglycerides, insulin and insulin resistance index (HOMA-IR), glycated hemoglobin (hemoglobin A1c or HbA1c), C-reactive protein (CRP), folate, and vitamin B12.

A hepatopathologist reviewed all of the liver biopsies and utilized the SAF scoring system (encompassing an assessment of steatosis (S), activity (A) and fibrosis (F)) scoring system for the grading and staging of steatohepatitis developed and validated by Bedossa and collaborators [16]. The staging, according to this scoring system, is as follows: steatosis: Stage 1—lipid droplets in ∼30% of hepatocytes, Stage 2—steatosis in 60% of hepatocytes and Stage 3—steatosis in >80% of hepatocytes; fibrosis: Stage 0—no fibrosis, Stage 1—zone 3 perisinusoidal fibrosis, Stage 2—as before with portal fibrosis, Stage 3—as before with bridging fibrosis, and Stage 4—cirrhosis; activity score: A0—no activity, A1—mild activity, A2—moderate activity, A3 (A ≥ 3)—severe activity).

### 2.3. Statistical Analysis

Before commencing any statistical processing and analysis, the data was visually inspected and assessed for outliers. Continuous variables were computed as the mean with standard deviation (SD), whereas categorical variables were expressed as percentages. Univariate and multivariate regression analyses were conducted using the vitamin levels as dependent variables. Rank correlation analysis was performed; both Spearman’s *rho* and Kendall’s tau coefficients were computed. Spearman’s *rho* coefficient was interpreted using the following rule of thumb (in absolute value): 0.00–0.19—very weak correlation, 0.20–0.39—weak correlation, 0.40–0.59—moderate correlation, 0.60–0.79—strong correlation, and 0.80–1.0—very strong correlation. Chi-squared test and analysis of variance (ANOVA)-one way were also performed.

All statistical analyses were performed with the commercial software Statistical Package for Social Science (SPSS version 24.0, IBM, Chicago, IL, USA). ROC analysis was performed with the commercial software MedCalc Statistical Software version 17.9.7 (MedCalc Software bvba, Ostend, Belgium; http://www.medcalc.org; 2017). A *p*-value of less than 0.05 was considered statistically significant.

## 3. Results

Eighty-three NASH patients were enrolled in this study. The mean age of the study population was 41.12 (SD ± 11.18) years. The majority of participants were male (61.4%). The mean serum levels of folate and vitamin B12 amongst the study population were 9.85 ± 10.90 ng/mL and 387.53 ± 205.50 pg/mL, respectively. Roughly half of the patients were presented with a grade 1 steatosis (43.4%), grade 2 fibrosis (50.6%) and a grade 3 activity score (55.4%). Further details regarding the study population can be seen in Table 1.

A statistically significant association was found between lower levels of folate and fibrosis grade on univariate regression analysis (*p*-value < 0.01). Older age, CRP levels, HOMA-IR, fibrosis grade and NASH disease activity grade were all significantly correlated with lower levels of vitamin B12 (Table 2).

When confounders such as obesity and insulin resistance were taken into account on multivariate analysis, fibrosis was found to be significantly associated with folate serum levels (standardized beta coefficient −0.353, *p* = 0.009). On rank correlation analysis, the link between folate levels and fibrosis grade was of statistical significance (Spearman’s *rho* −0.608 (95%CI from −0.728 to −0.451), *p*-value < 0.001, strong correlation; Kendall’s tau −0.487 (95%CI from −0.603 to −0.335), *p*-value < 0.001). Fibrosis was also significantly correlated with vitamin B12 levels, on both the regression (standardized beta coefficient −0.623, *p*-value = 0.000) and on rank correlation analysis (Spearman’s *rho* −0.737 (95%CI from −0.822 to −0.620), *p*-value < 0.001, strong correlation; Kendall’s tau −0.606 (95%CI from −0.688 to −0.489), *p*-value < 0.001). Activity was also associated with vitamin B12 levels (standardized beta coefficient −0.183, *p*-value = 0.039), even though the correlation found was very weak (Spearman’s *rho* −0.288 [95%CI from −0.474 to −0.0770), *p*-value = 0.0083; Kendall’s tau −0.237 (95%CI from −0.416 to −0.020), *p*-value = 0.0015). The analyses are presented in Table 3.

## 4. Discussion

Information regarding the relationship between folate and vitamin B12 status and the histological severity of NASH is scarce in the literature. In this study, both folate and vitamin B12 serum levels were found to be significantly correlated with the liver fibrosis grade, whereas vitamin B12 status was also associated with NASH degree of activity.

To date, the correlation between vitamin levels and chronic liver diseases has been investigated in several studies, although most of the data focused on vitamin D status [17,18,19]. Dasarathy et al. [20] compared plasma vitamin D concentration levels between 81 NAFLD/NASH patients, 67 individuals with hepatic steatosis and 39 healthy controls. Vitamin D serum levels were found to be significantly lower in NAFLD/NASH patients, with a negative correlation between vitamin D status and NAFLD activity scores. Low concentrations of vitamin D were additionally associated with greater severity of steatosis, hepatocyte ballooning, and fibrosis, although only the association with the severity of hepatocyte ballooning remained statistically significant after it was adjusted for confounders. Furthermore, plasma vitamin D and insulin concentrations were independent predictors of the NAFLD activity score on biopsy, while a higher fat mass was found to correlate with the low vitamin D levels in NAFLD patients. An additional study, which examined 46 morbidly obese patients with diabetes mellitus and metabolic syndrome, of which 72% had NASH [21], discovered a relationship between a higher fibrosis stage and higher levels of HOMA-IR and vitamin D. On the other hand, according to a study by Brill and colleagues [22], although subjects with NASH had higher insulin resistance than individuals without NASH, plasma vitamin D concentration was similar between both groups. However, despite these controversies concerning the role of vitamin D in NASH, other vitamins, such as folate and vitamin B12, have been overlooked in the extant literature.

Various studies have demonstrated that obese subjects have lower folate serum levels in comparison with normal-range BMI subjects [23,24]. Furthermore, obesity is a well-known risk factor for NAFLD [25]. Therefore, it has been suggested that folate deficiency may have a pathogenic role in NAFLD. This leads to a more severe form of the disease, mainly in metabolic syndrome and diabetes mellitus type 2 patients [26]. Moreover, Sid et al. [26] highlighted the importance of folate in the progression of NAFLD, thus raising the possibility that supplementary folate may be a treatment option. These findings are consistent with our results, in which a correlation between lower folate levels and a higher histological grading of NASH was revealed.

In a cohort of Chinese patients with NAFLD, low serum concentration of folate was identified as an independent risk factor for NAFLD [27]. Furthermore, it was demonstrated that inclusion of serum folate levels in the current NAFLD prediction score has led to a significant improvement in NAFLD prediction [27]. However, in an open-label pilot study [28], ten NASH patients with a median fibrosis stage 2 were treated with 1 mg/day of folic acid for 6 months. No significant effects in terms of biochemical improvement could be detected. Furthermore, folate deficiency was observed only in a cirrhotic patient. Therefore, it is clear that the controversy in the literature regarding the complex interplay between folate and non-alcoholic fatty liver disease and steatohepatitis has yet to be resolved. However, unveiling the pathogenetic process underlying the association between folate and NAFLD may provide important insights into the diagnosis and prediction of NASH, thus enabling treatment initiation in a timely and effective manner.

Another important finding of this study was the role of low vitamin B12 levels as an independent predictor of NASH histological severity, in terms of disease activity and fibrosis grade. These results corroborated the outcomes of previous studies, which, for instance, demonstrated that NAFLD patients have lower serum levels of vitamin B12 in comparison to controls, which correlated with a higher grade of steatohepatitis [29]. In another study [30], higher levels of homocysteine were observed in 71 NAFLD patients, which correlated negatively with folate and B12 concentrations.

However, in a study by Polyzos et al. [31], no difference in serum vitamin B12 and folate levels was detected in 30 NAFLD patients in comparison with 24 healthy controls. Additionally, no correlation was found between the levels of both vitamins and the severity of liver disease, in terms of steatosis grade and fibrosis stage. These results were in contrast with the findings of another study which reported that B12 could be used as a biomarker in a sample of 116 patients with chronic hepatitis C [32]. Serum B12 levels positively correlated with AST, ALT, baseline viral load, stage of fibrosis, and favorable interferon-λ3/4 rs12979860 genotypes. However, they were found to be inversely correlated with sustained virological response, as well as with rapid virological response.

Moreover, Goel et al. [33] found higher serum levels of vitamin B12 in patients with cryptogenic cirrhosis when compared to patients with idiopathic non-cirrhotic intrahepatic portal hypertension (NCIPH), therefore suggesting that B12 levels may be used as a differentiating marker between the two pathologies. Similarly, levels of vitamin B12 were also found to be significantly higher in patients with alcoholic liver cirrhosis than in matching healthy individuals [34]. Nevertheless, when taking into account the relevance of these results to the findings in our study, it should be noted that the majority (96%) of NASH patients in our study did not have cirrhosis. Therefore, it could be surmised that while low levels of vitamin B12 may predict the development of a severe form of NASH, high levels may indicate the commencement of end stage liver disease.

The relationship between serum vitamin B12 levels and insulin resistance has been widely addressed in the literature, yet remains controversial. Our study found that levels of vitamin B12 are inversely correlated with HOMA-IR, which is a known risk factor for NAFLD development [35]. This illustrates the probability that low serum levels of vitamin B12 might play a pathogenic part in NAFLD development. Similar findings regarding the ability of low levels of vitamin B12 and folate to predict insulin resistance were reported by Li et al. [36], however it should be noted that this study was conducted on obese subjects. Despite these compelling results, other studies did not detect a link between low vitamin B12 levels and insulin resistance or metabolic syndrome, whereas a correlation was found with obesity and being overweight [37]. Gammon et al. [38] reached an identical conclusion when assessing the relationship between insulin resistance and vitamin B12 levels in a vegetarian population. 

Our study has several limitations which ought to be properly addressed. The major drawback is the cross-sectional study design which, due to its very nature, does not enable the deduction of causal inferences with regard to the relationship between vitamin status and NASH in terms of development and pathophysiology. Another limitation is the lack of data concerning patient co-morbidities and drugs regimens. 

## 5. Conclusions

NASH is a chronic liver condition which imposes a dramatic burden, both in epidemiological and clinical terms. Our study demonstrated that low levels of folate (B9) and vitamin B12 can be used as independent predictors of the histological severity of NASH. Thus, these findings may have practical implications in the follow-up and prognosis assessment of NASH patients. Further studies regarding the pathological role of low folate and B12 levels in NASH development are warranted.

## Figures and Tables

**Table 1:** Descriptive statistics of the population studied.

Variables	Values
Age (years)	41.12 ± 11.18; 42 (18–74)
Gender	
Male	51 (61.4%)
Female	32 (38.6%)
BMI (kg/m^2^)	28.86 ± 2.83; 29 (22–40)
Fat volume	55.66 ± 24.60; 60 (10–90)
ALT (units)	80.61 ± 31.33; 78 (11–212)
AST (units)	48.81 ± 14.99; 45 (24–85)
CRP (mg/dL)	3.80 ± 2.44; 3.6 (0–13)
HDL (mg/dL)	37.69 ± 8.63; 36 (16–58)
LDL (mg/dL)	132.36 ± 24.01; 139 (68–187)
TG (mg/dL)	153.12 ± 36.63; 152 (69–274)
Insulin (IU/ML)	24.71 ± 5.21; 26 (12–36.2)
HbA1c (%)	5.38 ± 0.63; 5.4 (2–6.2)
HOMA-IR	2.88 ± 1.06; 2.8 (1.2–5.9)
B12 (pg/mL)	387.53 ± 205.50; 321 (123–823)
Folate (ng/mL)	9.85 ± 10.90; 8.9 (2.2–105)
Steatosis (S0–S3)	
S1	36 (43.4%)
S2	24 (28.9%)
S3	23 (27.7%)
Fibrosis (F0–F4)	
Grade 0	11 (13.3%)
Grade 1	20 (24.1%)
Grade 2	42 (50.6%)
Grade 3	7 (8.4%)
Grade 4	3 (3.6%)
Activity (A0–A4)	
Grade 2	36 (43.4%)
Grade 3	46 (55.4%)
Grade 4	1 (1.2%)

Abbreviations: BMI, body mass index; ALT, alanine transaminase; AST, aspartate aminotransferase; CRP, C-reactive protein; HDL, high-density lipoproteins; LDL, low-density lipoproteins; TG, triglycerides; HOMA-IR, homeostatic model assessment-Insulin resistance (HOMA-IR).

**Table 2:** Univariate regression analysis for each vitamin studied in the present investigation.

Variable	Folate (ng/mL)	B12 (pg/mL)
Age (years)	0.002 ± 0.108	−4.846 ± 1.971 *
Gender	2.730 ± 2.454	−2.138 ± 46.629
BMI (kg/m^2^)	0.085 ± 0.428	0.764 ± 8.062
Fat volume	0.008 ± 0.049	0.755 ± 0.924
ALT (units)	−0.034 ± 0.038	−1.291 ± 0.715
AST (units)	−0.090 ± 0.080	−1.972 ± 1.507
CRP (mg/dL)	−0.056 ± 0.500	−20.007 ± 9.072 *
HDL (mg/dL)	−0.105 ± 0.140	3.765 ± 2.612
LDL (mg/dL)	0.077 ± 0.050	−0.136 ± 0.951
TG (mg/dL)	0.032 ± 0.033	−0.722 ± 0.618
Insulin (IU/mL)	−0.431 ± 0.227	−7.564 ± 4.300
HbA1c (%)	−1.004 ± 1.908	−62.653 ± 35.350
HOMA-IR	−1.714 ± 1.127	−45.663 ± 20.947 *
Steatosis	−0.773 ± 1.450	30.421 ± 27.186
Fibrosis	−3.665 ± 1.218 **	−160.835 ± 16.351 ***
Activity	−3.196 ± 2.298	−131.579 ± 41.334 **

Abbreviations: BMI, body mass index; ALT, alanine transaminase; AST, aspartate aminotransferase; CRP, C-reactive protein; HDL, high-density lipoproteins; LDL, low-density lipoproteins; TG, triglycerides; HOMA-IR, homeostatic model assessment-insulin resistance (HOMA-IR). * statistically significant with *p*-value < 0.05; ** statistically significant with *p*-value < 0.01; *** statistically significant with *p*-value < 0.001.

**Table 3:** Multivariate regressions for each vitamin.

Variables	Non-Standardized Coefficients		Standardized Coefficients	T	Sig.
	B	Standard Deviation	Beta		
**Folate (B9)**					
(Constant)	6.959	21.122		0.329	0.743
Age (years)	0.188	0.128	0.193	1.469	0.147
Gender	5.101	2.710	0.229	1.882	0.064
BMI (kg/m^2^)	0.382	0.480	0.099	0.795	0.429
AST (units)	−0.087	0.095	−0.119	−0.914	0.364
ALT (units)	−0.003	0.107	−0.004	−0.031	0.975
TG (mg/dL)	0.024	0.035	0.079	0.680	0.499
HDL (mg/dL)	−0.147	0.148	−0.116	−0.996	0.323
LDL (mg/dL)	0.034	0.053	0.074	0.637	0.526
CRP (mg/dL)	0.519	0.567	0.116	0.915	0.364
HbA1c (%)	−0.065	2.113	−0.004	−0.031	0.976
Insulin (IU/mL)	−0.304	0.324	−0.145	−0.938	0.352
HOMA-IR	−0.742	1.697	−0.072	−0.438	0.663
Fibrosis	−4.079	1.513	−0.353	−2.695	0.009 **
Activity	−0.595	2.575	−0.028	−0.231	0.818
Steatosis	−6.073	3.461	−0.464	−1.755	0.084
Fat volume	0.188	0.120	0.424	1.566	0.122
**Vitamin B12**					
(Constant)	1264.063	280.796		4.502	0.000 ***
Age (years)	−0.814	1.705	−0.044	−0.478	0.634
Gender	−9.612	36.033	−0.023	−0.267	0.790
BMI (kg/m^2^)	−5.805	6.384	−0.080	−0.909	0.366
AST (units)	−0.586	1.260	−0.043	−0.465	0.643
ALT (units)	−2.413	1.416	−0.137	−1.704	0.093
TG (mg/dL)	−0.451	0.460	−0.080	−0.981	0.330
HDL (mg/dL)	3.060	1.961	0.129	1.561	0.123
LDL (mg/dL	0.547	0.702	0.064	0.780	0.438
CRP (mg/dL)	−5.564	7.537	−0.066	−0.738	0.463
HbA1c (%)	−31.261	28.088	−0.096	−1.113	0.270
Insulin (IU/mL)	1.503	4.304	0.038	0.349	0.728
HOMA-IR	−4.149	22.558	−0.021	−0.184	0.855
Fibrosis	−135.886	20.118	−0.623	−6.754	0.000 ***
Activity	−72.172	34.236	−0.183	−2.108	0.039 *
Steatosis	66.491	46.012	0.270	1.445	0.153
Fat volume	−1.966	1.594	−0.235	−1.233	0.222

*Abbreviations:* BMI, body mass index; ALT, alanine transaminase; AST, aspartate aminotransferase; CRP, C-reactive protein; HDL, high-density lipoproteins; LDL, low-density lipoproteins; TG, triglycerides; HOMA-IR, homeostatic model assessment-Insulin resistance (HOMA-IR). * statistically significant with *p*-value < 0.05; ** statistically significant with *p*-value < 0.01; *** statistically significant with *p*-value < 0.001.
